# Effect of Virtual Zen Garden on Quality of Life of Residents in Long-Term Care Home

**DOI:** 10.3390/ijerph22040510

**Published:** 2025-03-27

**Authors:** Ivo Yuen, Timothy Kwok

**Affiliations:** 1 Department of Medicine and Therapeutics, Faculty of Medicine, The Chinese University of Hong Kong, Hong Kong 999077, China; 2 Jockey Club Institute of Ageing, The Chinese University of Hong Kong, Hong Kong 999077, China

**Keywords:** wellbeing, older persons, virtual reality, Zen Garden, long-term care

## Abstract

Increasing attention has been paid to the therapeutic effect of gardens on older persons in long-term care homes. However, problems on set up and reliability of implementation were still of concern in studies. This study investigated residents’ experiences of Virtual Zen Garden in Hong Kong. Twenty-four residents were interviewed using semi-structured interviews based on the phenomenological research approach in two long-term care homes. This study aligns with the COREQ (Consolidated Criteria for Reporting Qualitative Research Checklist). Nine theme clusters were organized that described the influence of Virtual Zen Garden on residents’ quality of life in long-term care homes. They are ‘staying engaged despite no change in physical condition’, ‘embracing current physical functioning’, ‘being the one to exercise autonomy’, ‘fostering a sense of relaxation and acceptance to the past, current and future lives’, ‘fostering a sense of satisfaction, purpose and mastery’, ‘open to companionship with staffs’, ‘feeling a sense of connection with other residents’, ‘perceiving a sense of security and identity in the living environment’ and ‘accepting the place they stay as a home in the rest of life’. Virtual Zen Garden demonstrated positive beneficial effect on quality of life in physical, psychological, social, and environmental perspectives. The findings were encouraging for the introduction of Virtual Zen Garden as an innovative intervention, into long-term care homes.

## 1. Introduction

The worldwide proportion of older persons is continuously increasing. Older persons commonly suffer from a gradual decline in psychological functions, multi-morbidity, and inability to perform activities of daily living [1,2,3,4]. Consequently, the demand on long-term care homes has been increased consistently. In the United States, there were 15,300 long-term care facilities accommodating 1,294,800 residents in 2020 [5]. The ratio of institutional placement to population of older person in China was 1:34 in 2022 [6]. Owing to poor health, fragility and lack of independence of residents, it is not uncommon that they spend their time in their rooms, which can lead to lack of stimulation, isolation, and decreased socialization [7]. As a result, increasing attention has been paid to the consequent quality of life of residents in long-term care homes.

Quality of life is a broad and complex concept influenced by physical, spiritual, and social situations of individuals, personal, faith, as well as relationship with the environment [8]. A variety of interventions have been developed to promote quality of life in long-term care home residents. One of these interventions is the use of garden [9,10,11]. Quantitative studies showed small yet consistent effect of outdoor garden as a multisensory environment on improving affect and wellbeing in older persons [12,13,14]. For examples, horticultural therapy and other person-centered garden activities such as sitting, walking, having a conversation, drinking a beverage, or having lunch [15]. Given that there is a limitation of space, recent studies have begun to put emphasis on the use of virtual reality to evoke some of the beneficial effects of garden in a more feasible way [16,17,18]. While virtual representation of nature yielded a range of effect, studies on effect of virtual garden on quality of life is limited [19,20].

Virtual reality resembles real-life situations in which users can have close-to-reality experiences [21]. Virtual reality can be immersive and non-immersive, with the level of immersion allowing a sense of presence in the environment [22]. Past studies have shown that both immersive and non-immersive virtual nature are effective, for older persons living in both community and long-term care homes, in reducing pain, stress, heart rate, and blood pressure while they can increase restoration, vitality, and improve emotion [23,24,25,26,27,28,29,30]. These effects are often attributed to two influential theories, namely the attention restoration theory and the stress recovery theory [31,32,33]. The attention restoration theory proposes that voluntary attention depletes in urban environments and during cognitively demanding tasks, while it is restored in natural environments [34]. The stress recovery theory claims that natural environments influence affective states, hence, facilitating recovery from stressors [35]. However, virtual reality experience and its therapeutic effect are affected by the design of content being represented, which might lead to unwanted effects such as motion sickness and boredom [20,36]. Therefore, careful consideration on the type and design of virtual garden is needed to optimize its effectiveness.

Zen Garden from Asian culture is specifically developed to calm one’s mind by viewing miniaturized scenery [37]. The word ‘Zen’ signifies its use in meditation. For example, the world-famous Zen Garden is composed with only fifteen small stones on white sand, and it is used by practitioners of meditation in the Ryoan-ji Temple [38]. Previous studies have shown that viewing Zen Garden built in a confined space improves the quality of life of older persons [37,39,40]. Unlike other gardens that facilitate physical interaction with the elements of nature, Zen Garden requires no direct physical contact with, or movement of person within the garden [41,42,43]. It is a pictorial representation of nature to be viewed from certain specific viewing points. Viewers being still in the process gives Zen Garden the advantage of minimizing the unwanted effect of motion sickness when being virtually represented. Therefore, Zen Garden can be readily transformed to a virtual intervention on improving quality of life.

The design of Zen Garden emphasizes the principles of naturalness, simplicity, and austerity. The sparse and seemingly random composition of rocks and empty rectangle of raked gravel form a fascinating landscape. A good design of Zen Garden is based on two aspects [44]. One is the spatial structure that refers to the perceptual attributes of stimuli. While the rocks have irregular and asymmetrical shapes, natural patterns formed by them are often self-similar and contribute to implicit regularity of Zen Garden [45]. Another aspect of good design is the integrated impression that refers to the synthesized evaluations made by viewers. The rocks abstractly represent natural elements such as islands in the ocean, mountain top above the clouds or strokes of the Chinese character meaning ‘heart’ or ‘mind’ [46]. A well-designed Zen Garden induces a sense of harmony and imagination during the inexhaustible process of engagement. These distinctive features should be incorporated when developing Virtual Zen Garden.

Virtual Zen Garden is a virtual representation of Zen Garden that has potential to improve quality of life of residents in long-term care homes. A feasibility study has shown that residents in long-term care homes were satisfied with the Virtual Zen Garden and its positive benefit on quality of life, emotion, and social relationship [47]. Compared with another study on virtual vegetable garden in Taiwan, which evoked positive emotions by stimuli related to participants’ experience of gardens in the past, the effect of Virtual Zen Garden was less dependent on viewers’ characteristics [19]. This suggests latent factors contributing to the consistent effect of Virtual Zen Garden on residents with various background in long-term care homes. Further exploration is needed to address the knowledge gap of Virtual Zen Garden in terms of its extent of influence and contribution of its design on improving quality of life. The present study aimed to explore the process and experience of residents in Virtual Zen Garden in long-term care homes. It also aimed to gain an in-depth understanding on how Virtual Zen Garden influences on their quality of life in terms of physical, psychological, social, and environmental perspectives.

## 2. Material and Methods

### 2.1. Study Design

A qualitative exploratory descriptive design was adopted. Semi-structured interviews were conducted after a randomized controlled trial of Virtual Zen Garden (n = 160) in two long-term care homes in Hong Kong. A phenomenological research approach was used to understand the experience of participants in Virtual Zen garden and its influence on their quality of life. Informed consent was obtained from residents without dementia and guardian of residents with dementia before data collection started. A diagnosis with dementia according to DSM-IV or DSM-V or demonstrated impairment in cognition according to the Mini-Mental State Examination (cutoff points for dementia vary from 18 in illiterate participants to 22 for those with more than 2 years of education) was used to confirm participants with dementia [48,49,50].

### 2.2. Participants

Purposive sampling was adopted to capture the experience of the phenomenon under investigation [51]. After the randomized controlled trial of Virtual Zen Garden, participants aged 65 or above and with length of residence more than 3 months were considered eligible if they completed intervention period with full attendance and were able to respond accurately in semi-structured interview [52]. These attempted to elicit more vivid response from the eligible participants during interviews [47]. Based on the consideration of the degree of repetition of data and the depth of understanding on the influence of Virtual Zen Garden on quality of life, sampling from both the intervention group (IG) and the control group (CG) continued until data were saturated, ranging from 5 to 15 in phenomenological studies involving interviewees with and without dementia [53]. A total of 24 participants were recruited (12 participants from IG and 12 participants from CG).

### 2.3. Intervention Group—Virtual Zen Garden

**Figure 1:**
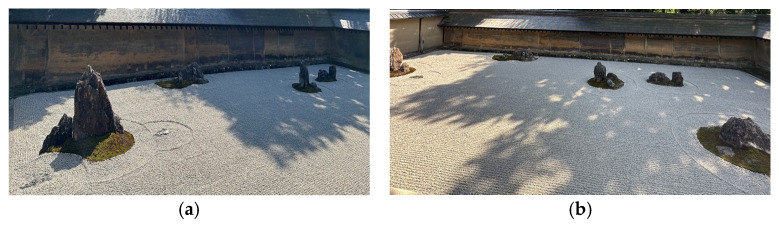
The examples of Zen Garden virtually represented in IG. (**a**) The side view; (**b**) the front view.

The aims of Virtual Zen Garden intervention are to foster soothing and sensorial environment to improve quality of life of participants through viewing the landscape of Zen Garden together with a nature sound. The visual image and sound were recorded from the Zen Garden in the Ryoan-ji Temple. It consisted of 24 bi-weekly group sessions, each lasting for 60 min, for 3 months. Equipment including a projector and speakers, and 110-inch diagonal 16:9 screens were set up in a 700 square feet room to simulate Zen Garden. In addition, participants’ level of anxiety and depression were monitored before and after intervention using the rating anxiety in dementia scale and the Cornell scale for depression in dementia [54,55]. Components of Zen Garden include arrangement of rocks, sand, and gravel pattern. In each session, Zen Garden was presented as a set of still photos each following in order of landscape as a whole, followed by components of landscape and then the repeated overall landscape. There were 6 sets presented in total with each set lasting for 10 min. The examples of Zen Garden virtually represented in IG are presented in Figure 1a,b.

### 2.4. Control Group

**Figure 2:**
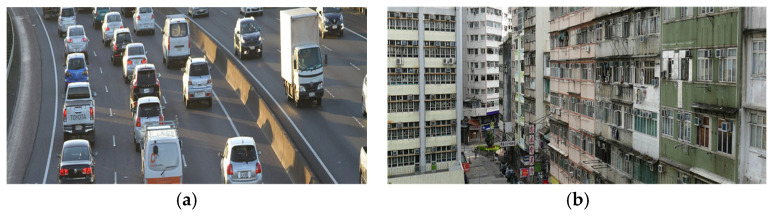
The examples of urban scenes virtually represented in CG. (**a**) The traffic scene; (**b**) the city block scene.

In order to control the social effect on quality of life on participants, the control group adopted the same equipment and duration and frequency of session as the intervention group. In each session, urban scenes together with traffic sound were presented. Components of urban scenes include city blocks, roads, and traffic. In each session, the order of presentation of urban scenes was similar to that in intervention group. The examples of urban scenes are presented in Figure 2a,b.

### 2.5. Data Collection

Data collection was conducted from 1 July 2023 to 31 July 2023. A semi-structured interview was conducted on an individual basis to explore participants’ perception and experience in the trial. The duration of interview was between 17 and 25 min to maintain data quality with consideration of age and cognitive function of interviewee [56]. All interviews were audio recorded, and the recorded content were then transcribed verbatim by a research assistant. After that, the researcher confirmed the correctness of the content by comparing the audio recordings with the transcript.

The semi-structured interview guide was developed, which consisted of open-ended questions with introductory, transition, main, and closing questions. After starting the introductory questions with greetings and questions regarding the experiences in sessions of either IG or CG, the interviewer naturally approached the research question using the following transition question: ‘How would you describe your quality of life?’. After that, the interviewer asked the main questions such as ‘How did the virtual experience affect your mood?’, ‘Have you noticed any changes in your physical health or energy levels since participating in the sessions?’, ‘Has the virtual experience impacted feelings of loneliness or isolation?’, and ‘How would you describe your feeling toward your living environment?’. Finally, the interview was concluded with an open question asking for additional opinions. During the interview, participants were given ample time and opportunity to share their experiences and opinions. The interview continued until no further comments were made. Any ambiguous statements from participants were clarified through additional questioning.

### 2.6. Data Analysis

The seven steps of Colaizzi’s method were followed in this study. In brief, this method consists of seven steps: (i) to read and re-read each of the transcripts in order to obtain a general sense about the whole content; (ii) to extract significant statements that pertain to the phenomenon under study; (iii) to formulate meanings from these significant statements; (iv) to organize the formulated meanings into themes, cluster of themes, and categories; (v) to integrate the findings into an exhaustive description of the phenomenon under study; (vi) to describe the fundamental structure of the phenomenon; (vii) to validate the findings with the study participants to compare the descriptive results with their experiences [57]. The interview transcripts were analyzed by a principal investigator and coordinator of qualitative data collection. The quotes, codes, and themes were translated into English, then back translated into Chinese by independent translators.

### 2.7. Data Quality

Several measures were taken to enhance the trustworthiness of the study [58]. First, the principal investigator and coordinator of qualitative data collection were allied health professionals in the study settings with experience of more than 5 years. Therefore, they had the chance to familiarize themselves with the local culture. Second, the interviews were conducted at the interviewees’ place of convenience such as bedroom and dining room. Initial chatting helped to foster casual conversation before starting the interview. Third, interpretations of transcribed interviews were discussed among six members of qualitative study team to reduce possible bias. The process of study aligns with consolidated criteria for reporting qualitative research (COREQ). A pilot study lasting for 6 months had been conducted to justify the use of interview guide by trained interviewers, procedure of the study and the process of translation by bilingual translators [47].

### 2.8. Ethcial Approval

Ethical approval was granted for this study by the Survey and Behavioural Research Ethics Committee of the Chinese University of Hong Kong (SBRE-21-0819). Data were anonymized at the point of transcription.

## 3. Results

A total of 24 participants were interviewed (4 males and 8 females from each group, with an average age of 87.46 years). After data collection, there were 281 and 178 meaningful statements derived from IG and CG, respectively. Through repeated verification with relevance to the study’s topic, there were 29 and 19 themes composed from IG and CG, respectively. According to the similarity of meanings, data were organized into nine theme clusters and structured into four categories (Table 1).

### 3.1. Category 1: Influence on Physical Wellbeing

Participants in the IG reported a different influence on physical wellbeing when compared with those in the CG. The category in the IG was composed of ‘staying engaged despite no change in physical condition’ and ‘embracing current physical functioning’, which is contrary to that in the CG, which was composed of ‘participation being discouraged by physical condition’ and ‘rejecting self in view in perspectives of physical condition and physical functioning’.

#### 3.1.1. Stay Engaged Despite No Change in Physical Condition

Participants in IG stated that they were able to sustain their attention beyond their expectation and endure on pain and numbness. As a result, they were able to find beauty in Virtual Zen Garden amidst suffering. Alternatively, participants in the CG reported of struggles for normalcy and feeling of powerless in the presence of pain.

‘My body remains largely unchanged, except for the persistent presence of pain… No notable alterations have occurred… I can (only) concentrate on watching TV undisturbed, as nobody interrupts me’.

(CG)

‘The pain continues to pervade my entire body, yet I choose to release its hold on me… Despite the numbness in my hands, I am able to behold the landscape before me…’.

(IG)

‘I had not anticipated staying awake during the session, but that’s exactly what happened… The scene appeared simple at first, but upon taking a more detailed look, I noticed an array of rocks with unique shapes and circular patterns on the ground, they were truly captivating…’.

(IG)

#### 3.1.2. Embracing Current Physical Functioning

Participants in IG showed opposing attitudes towards their physical functioning compared with those in the CG. After the intervention of Virtual Zen Garden, participants reported of making use of their residual ability to cope with limitations in mobility, and they were satisfied with the decreasing choice of activity as they aged. In addition, they showed acceptance on the change in the way of performing daily tasks and the current level of assistance. In contrast, participants in the CG felt disappointed and useless towards their mobility and decline in physical tolerance.

‘Due to my weakness in lower limb, I used to have to put in a lot of effort to go out. However, now I can enjoy the landscape without stepping outside… The way I live today is distinct from the past when I possessed more energy and could engage in work. Presently, it proves quite challenging for me…’.

(CG)

‘Despite relying on a wheelchair and having limited mobility, I can still access this place and delight in its scenic views… If I experience discomfort from sitting for extended periods, I have the flexibility to adjust my posture, allowing me to continue observing the scenes…’.

(IG)

‘Engaging in activities as I did in the past becomes increasingly challenging due to my age. However, presently, sitting and passing time proves to be satisfying enough for me… Each person possesses their unique strengths and weaknesses… There is no need to compare oneself with others. It is perfectly acceptable to seek assistance from others when needed’.

(IG)

### 3.2. Category 2: Influence on Psychological Wellbeing

Participants in the IG were found to be influenced differently in psychological wellbeing when compared to those in the CG. The category in the IG comprised ‘being the one to exercise autonomy’, ‘fostering a sense of relaxation and acceptance to the past, current and future lives’ and ‘fostering a sense of satisfaction, purpose and mastery’. The category in CG comprised ‘denying on not being able to make choice’, ‘inducing a sense of tension’, and ‘ambiguating the purpose of life’, which were contrasting to the respective clusters of themes in the IG.

#### 3.2.1. Being the One to Exercise Autonomy

Participants in IG had developed the senses of self-control, empowerment, dignity, and respect. They perceived themselves as being able to make choices and becoming unique. However, participants in the CG revealed thoughts of obeying the staff’s arrangement as a kind of freedom, which rationalized their reliance on staff for participation.

‘Participating in activities signifies that I am still capable of accomplishing tasks… Being invited by others (staffs) signifies that I have the freedom to choose’.

(CG)

‘I have the ability to train myself to maintain stillness and exert control over my body… I continue to find solace in nature, appreciating its wonders in my own company…’.

(IG)

‘Despite being unable to control my surroundings and the actions of others, I retain the power to determine my own actions… I have the potential to be unique, much like stones with diverse shapes… It all comes down to the choices I make…’.

(IG)

#### 3.2.2. Fostering a Sense of Relaxation and Acceptance to the Past, Current and Future Lives

As a result of receiving intervention of Virtual Zen Garden, participants felt calm and peaceful and not being troubled by the past. In addition, they were open to companionship and expressed a more positive attitude towards their current and future lives with satisfaction and hope. However, participants in the CG struggled with a mix of emotions, and they put effort into masking their emotions in the sessions. Their struggles were also complicated by their desires for companionship and the feeling of isolation.

‘I felt immense joy when someone extended an invitation for me to partake in this exceptional program… Individuals are more prone to boredom compared to a collective group of people… Despite venturing into new experiences, I remained calm and composed. The process felt manageable and acceptable…’.

(CG)

‘An overwhelming sense of tranquility washed over me as I beheld the patterns formed by the stones and gravel’.

(IG)

‘An overwhelming sense of tranquility washed over me as I beheld the patterns formed by the stones and gravel… At that moment, I experienced a profound sense of relaxation, liberated from the shackles of the past… Simply keep living, and everything will be alright… I consider myself fortunate to reside in this place, where even the stacks of stones are transformed into objects of beauty. It reminds me that I have the power to create an incredible life…’.

(IG)

‘When engaged in the same activity and observing shared scenes, the barriers between people dissolve… As I opened myself up to them, I noticed a distinctiveness in others. They exuded a peaceful and friendly demeanor…’.

(IG)

#### 3.2.3. Fostering a Sense of Satisfaction, Purpose and Mastery

During the session of Virtual Zen Garden, participants experienced the sense of satisfaction and achievement. These senses originated from their perceived control over time in the session and experience in meditation. Moreover, they put value on choosing tasks to create purpose of life. While participants in the CG were expressed that they were satisfied by treating indifferently to follow schedule for non-specific activities, they showed ambiguous pursuit of relief from boredom.

‘I sensed a greater fulfilment in my life as I established a regular schedule for participating in activities… When I have tasks or activities to occupy my time, I don’t experience boredom…’.

(CG)

‘It appears that engaging in meditation brings about a sense of satisfaction… I can establish my own rhythm to fully immerse myself in the surroundings. Everything aligns with my own guidance… I deliberately slowed down my pace and, to my surprise, discovered that it was quite comfortable to gaze at the screen… I came to the realization that I am capable of paying attention to such a degree. I discovered my own worth and now have a desire to continue participating in this activity to further explore my potential…’.

(IG)

### 3.3. Category 3: Influence on Social Relationship

During the sessions, similar and different reflections on social relationship were noted in the IG when compared with the CG. This category in the IG consisted of ‘open to companionship with staffs’ and ‘feeling a sense of connection with other residents’, while this in the CG consisted of ‘thinking of inequality in relationship with staffs’ and ‘building connections through shared experiences’.

#### 3.3.1. Open to Companionship with Staff

Participants in the IG reflected on a deeper emotional bonding formed by daily interaction and genuine concern from staff while participants in CG reflected on power imbalance and dependency in their relationship with the staff. The narratives of participants in both the IG and CG revealed their positive attitude towards caring staffs while mutual support in the resident–staff relationship was only reflected in the IG, which was indicated by participants’ commitment to be considerate and respectful of staffs’ effort in care.

‘To venture outside, I rely on staff members for assistance. Otherwise, I am confined to my room… They provide invaluable assistance by supporting my body and preparing my meals. There are times when I feel groggy and reluctant to leave my bed, but they patiently guide me to hold onto the handles and place my feet gently on the floor’.

(CG)

‘We share an emotional connection with the staff members as we are in the same location and interact with each other on a daily basis… The atmosphere becomes more pleasant when they are present here. Occasionally, we engage in small talk, and their genuine concern for me shines through’.

(IG)

‘They are kind individuals who take care of my daily needs and accompany me in participating in activities… I must make an effort to alleviate the workload of the staff members who are here to assist me’.

(IG)

#### 3.3.2. Feeling a Sense of Connection with Other Residents

Both participants in the IG and CG showed a change on perception towards other residents in the sessions. Participants in the IG attributed this change to the shared experiences of tranquility, while those in the CG attributed this change to their initiative to build up relationship with other residents. Although participants in the CG superficially reflected that shared experience could strengthen their connection with other residents, they were still exploring the ways to strengthen their bonding without elaborating on the kind of activities providing such experience. Only participants in the IG showed acceptance on diversity among residents and related their indivisible bonding as part of nature.

‘Others appeared friendlier and more approachable than usual. We were at least willing to sit together in each other’s company… Our sense of connection deepens when we engage in shared activities…’.

(CG)

‘I observed others embracing a sense of calmness, attentively listening to the sound of cicadas and gazing at the stones. In that moment, we grew closer to one another and experienced a comforting atmosphere… When I witnessed their serene and composed demeanor, my perception of them changed. Previously, they would roam around, intrude into others’ rooms, and were challenging to approach. However, now I have adopted a calm and considerate approach when they enter my room’.

(IG)

‘It is a twist of fate that individuals from various families find themselves living together here… We are inseparable, as we are all integral parts of nature…’.

(IG)

### 3.4. Category 4: Influence on Environemntal Domain of Quality of Life

Participants in the IG and the CG perceived a sense of security yet with different reasons, and they showed different attitudes towards a place being a home. This category in the IG consisted of ‘perceiving a sense of security and identity in the living environment’ and ‘accepting the place they stay as a home in the rest of life’ while this in the CG consisted of ‘perceiving a sense of security and longing for active participation’ and ‘preferring a place to be a home when staying with family’.

#### 3.4.1. Perceiving a Sense of Security and Identity in the Living Environment

Both participants in the IG and CG felt secure due to care service in long-term care homes. In contrast to the CG, participants in the IG showed deeper reflection on reciprocal acceptance, personal growth and the sense of belonging. Therefore, they anchored their identity to their living environment.

‘The presence of nurses and care workers who look after us instills a sense of peace, even during times of illness… I eagerly desire to engage in activities and actively participate in the life here’.

(CG)

‘The staff members are thoughtful and mindful, ensuring that I am kept safe and protected from harm… I feel embraced and acknowledged as someone who requires care, which allows me to live without undue concerns. In the same way, I also extend acceptance to others and the things within this environment’.

(IG)

‘I am a valued member of this residence, where I have ample time to find inner tranquility… I sensed a profound connection with my inner self, gradually experiencing a deep sense of peace. Reflecting on my journey of adapting to this place, I believe it has become the destination of my life’.

(IG)

#### 3.4.2. Accepting the Place They Stay as a Home in the Rest of Life

Participants in the IG achieved a definition of home from the sense of comfort, respectful relationships, and stability of their living environment, which were in contrary to those in the CG who emphasized the importance of family. Therefore, participants in the IG showed stronger emotional attachment to their living environment than those in the CG.

Most participants mentioned that home is a place for family living there. Some participants in Virtual Zen Garden considered a place as home by the feelings during their interaction with others and surroundings rather than by the presence of specific member. They mentioned that home is a place providing comfort and people living there respect each other.

‘A place can be deemed a home when it is comprised of family members. Otherwise, it does not hold the same essence… I prefer to remain here, as it provides me with a structured routine encompassing meal times, bathing time, and bedtime. That’s all I need for my day’.

(CG)

‘I believe that any place can become my home if it provides comfort and the people residing there treat one another with respect… I desire for this place to be my final abode, where I have no need to relocate. I wish to stay here for as long as I am able’.

(IG)

## 4. Discussion

This study revealed the influence of Virtual Zen Garden to quality of life in terms of physical wellbeing, psychological wellbeing, social relationship, and environmental domains. In comparison to the control, Virtual Zen Garden elicited more and deeper reflection of participants from which more themes were yielded. When comparing the themes between IG and CG, participants who experienced Virtual Zen Garden were more able to stay engaged. They were also easier to perceive the senses of relaxation, acceptance, self-control, empowerment, dignity, satisfaction, purpose, and mastery. In addition, they showed more emotional attachment upon their reflections towards staff, residents, and their living environment. As a virtual reality intervention, Virtual Zen Garden has some of the beneficial effects on quality of life compared with the real garden intervention [59]. Furthermore, no unwanted effects such as motion sickness and boredom were reported by participants in this study [20,36]. These suggest that Virtual Zen Garden is effective for improving older persons’ quality of life, and it offers a feasible option of intervention in the presence of limited space in long-term care homes [16,17,18]. Whereas a previous study on Virtual Zen Garden focused on its development [47], this study was the first to attempt to relate its design with the effect on improving quality of life.

That Virtual Zen Garden itself represents a scene of nature and contributes to its effect that is similar as viewing nature. The findings on the engagement process and sense of relaxation support that the effect of Virtual Zen Garden is in line with the attention restoration theory and stress recovery theory [31,32,33]. The findings also indicate that the design of Virtual Zen Garden creates the sense of being away and fascination. These are two mediators to the effect of garden on enhancing self-perceived health of older persons in long-term care homes [60]. The virtually represented patterns of Zen Garden formed by natural placement of rocks over a plain background contribute to the viewer’s impression of nature view and probably with greater width and depth [61]. In addition, the pattern of Zen Garden and asymmetry of rocks in the whole scene contribute to unlimited spectacles in natural scene by just a little change in viewing position [61]. While Virtual Zen Garden utilizes the unique spatial structure of Zen Garden in creating and enhancing the effect of viewing nature, the impression and subsequent response of viewers link its design to improvement in quality of life.

The symbolism of Zen Garden, manifested in its virtual representation, engages the imagination of its viewers, which was often noted among statements of participants in this study. This process is implied in the underlying philosophy of the design of Zen Garden for practitioners of meditation to achieve a state of intrinsic enlightenment. In contrast to the strong references to individualism and humanism in shaping the design of Western gardens, the intent of Zen Garden is to be a representation of the larger world of nature instead of a symbol of a human activity [19]. Its purpose, therefore, is to extend a deep veneration of nature and a unique fascination with and adaptation to the complex relationship between the natural world and mankind. The symbolism is a unique feature of Zen Garden, which helps explain its extensive effect over different domains of quality of life in this study. However, further exploration on the mechanism of symbolism for improvement in quality of life is needed.

There are several limitations of this study. First, although the sample size was low, participants were recruited from two long-term care homes until data saturation was reached, at which point our latter interviews did not yield additional insights into the influence of the intervention on quality of life. This approach is acceptable for qualitative studies that aim to gain an in-depth understanding of a concept from a group sharing similar properties. However, caution is still required in interpreting the findings. Second, recruitment of participants who fully completed the intervention period of Virtual Zen Garden may produce selection bias in favor of interviewees attracted by this kind of virtual garden intervention. Consequently, some influences are likely to be overrepresented and were depicted in great detail. Despite the restriction of data collection results in limitations for the transferability of findings, studies focusing on the user perspective is necessary to the development of intervention for improving quality of life. Third, age and sex are two further factors that created a certain bias. As participants were recruited from long-term care homes, the majority of them were older than 83 years. Findings revealing the perceptions of older persons may be subjected to influence of age decline in recognition of negative information and greater engagement with highly positive emotional context in older persons [62,63]. Meanwhile, females dominate among interviewees, and the findings demonstrate the needs of females more so than males. This gender bias might be a result of the longer life expectancy of females compared with males. They are consequently more likely to experience life events such as widowhood and institutionalization that diminished their quality of life. Their report revealed the influence of Virtual Zen Garden on adaptation to life events and restoration of quality of life. However, perceptions of older persons who were younger and males remain underrepresented in this study.

## 5. Conclusions

The interviews indicated the experience of residents in Virtual Zen Garden and its influence on quality of life. Virtual Zen Garden demonstrated a positive beneficial effect on quality of life in physical, psychological, social, and environmental perspectives when compared with the control. The participants remain actively engaged in life and embrace their current physical functioning. Receiving intervention of Virtual Zen Garden fostered the senses of relaxation, acceptance, self-control, empowerment, dignity, satisfaction, purpose, and mastery. Virtual Zen Garden also facilitated deeper reflection of participants on their social relationship and living environment, which contributed to the senses of connection, security, and identity. The findings were encouraging for introduction of Virtual Zen Garden, as an innovative intervention, into long-term care homes. This offers a possible means of easy and reliable delivery of virtual nature interventions in long-term care homes.

## Figures and Tables

**Table 1:** Influence of virtual experience on quality of life in intervention group (IG) and control group (CG).

Category	Theme Cluster		Theme (Number of Participants Who Produced Material Included Within Each Theme)	
	IG	CG	IG	CG
Influence on Physical Wellbeing	Staying engaged despite no change in physical condition	Participation being discouraged by physical condition	Endures on pain and numbness (9)	Struggles for normalcy amidst pain (10)
			Finds beauty in Virtual Zen Garden amidst suffering (9)	Feels powerless to persistent pain (10)
			Sustains attention beyond expectation of self (12)	
	Embracing current physical functioning	Rejecting self in view of decline in physical functioning	Makes use of residual ability to cope with limitations in mobility (9)	Feels disappointed on current mobility (9)
			Is satisfied with choices of activities decreasing with age (9)	Feels useless in view of decline in physical tolerance (9)
			Accepts the change in the way of doing daily tasks and current level of assistance (10)	
Influence on Psychological Wellbeing	Being the one to exercise autonomy	Denying on not being able to make choice	Develops a sense of self-control (9)	Relies on staffs for participation (10)
			Develops a sense of empowerment to make choice and become a unique person (9)	Deceives self that obeying on staff’s arrangement is a kind of freedom (9)
			Develops a sense of dignity and respect (10)	
	Fostering a sense of relaxation and acceptance to the past, current and future lives	Inducing a sense of tension	Feels calm and peaceful (12)	Experiences a mix of emotions (9)
			Is not troubled by the past (10)	Puts effort on masking emotions of self (9)
			Is satisfied with current situation (10)	Desires for companionship, yet feeling isolated (10)
			Feels hopeful for the future (9)	
			Is open to companionship (10)	
	Fostering a sense of satisfaction, purpose and mastery	Ambiguating the purpose of life	Feels satisfied with the sense of control over time (10)	Becomes satisfied by treating indifferently to follow schedule for non-specific activities (10)
			Feels sense of achievement in meditation and desires to further explore the potential of self in meditation (9)	Desires for being occupied by non-specific tasks to relieve boredom (10)
			Values on tasks chosen by self to create purpose of life (9)	
Influence on Social Relationship	Open to companionship with staffs	Thinking of inequality in relationship with staffs	Reflects on emotional bonding with staffs built up from daily interaction and genuine concern from staffs (9)	Reveals power imbalance and dependency in the relationship with staffs (9)
				Appreciates the one-sided effort of staffs in care (9)
			Shows positive attitude towards caring staffs (12)	
			Shows commitment to be considerate and respectful of staff’s effort in care (10)	
	Feeling a sense of connection with other residents	Building connections through shared experiences	Reflects on a shift in perception and relationships with other residents that occurs through shared experiences of tranquility (10)	Shows initiative to build up relationship with other residents (9)
				Thinks on exploring the ways to strengthen the bond with other residents (8)
			Embraces unity in diversity among residents (9)	
			Reflects on indivisible bonding with other residents (9)	
Influence on Environmental domain of quality of life	Perceiving a sense of security and identity in the living environment	Perceiving a sense of security and longing for active participation	Reflects on reciprocal acceptance, care and support in the living environment (10)	Feels secure in presence of illness due to care services (10)
			Feels secure for personal growth and reflection (9)	Unable to fulfil the desire to get involved in the living environment (9)
			Feels sense of belonging to living environment (9)	
			Anchors identity to living environment (10)	
	Accepting the place they stay as a home in the rest of life	Preferring a place to be a home when staying with family	Defines home from the feeling of comfort, respectful relationships and stability of living environment (10)	Emphasizes the importance of family in defining home (10)
			Shows a strong emotional attachment to the living environment (9)	Shows avoidance on forming deeper emotional attachment to the living environment (9)

## Data Availability

The original data presented in this study are included in the article. Further inquiries can be directed to the corresponding author.
